# Circulating 1,5-anhydroglucitol levels combined with pre-pregnancy BMI is a simple predictive marker of future glucose intolerance in women with gestational diabetes

**DOI:** 10.1007/s40618-025-02768-1

**Published:** 2025-12-12

**Authors:** Dan Yedu Quansah, Cecilia Jiménez-Sánchez, Pierre Maechler, Jardena J. Puder

**Affiliations:** 1 Obstetric service, Department Woman-Mother-Child, Lausanne University Hospital, University of Lausanne, Lausanne, Switzerland; 2 Department of Cell Physiology and Metabolism, University of Geneva Medical Center, Geneva, 1206 Switzerland; 3 Faculty Diabetes Center, University of Geneva Medical Center, Geneva, 1206 Switzerland; 4 Faculty of Biology and Medicine, University of Lausanne, Rue du Bugnon 21, Lausanne, CH-1011 Switzerland

**Keywords:** 1,5-Anhydroglucitol, Gestational diabetes, Glucose intolerance, HOMA-IR, Insulin secretion and resistance, Adjusted insulin secretion, Postpartum

## Abstract

**Introduction:**

In women with gestational diabetes (GDM), 1,5-Anhydroglucitol (1,5-AG) levels are lower compared to controls. However, data on 1,5-AG changes in the postpartum and its associations with indices of insulin secretion or resistance in the perinatal period are lacking. This prospective study assessed the trajectory of 1,5-AG, 1,5-AG/HOMA-IR and 1,5-AG/BMI during pregnancy up to 1-year postpartum, their association with indices of insulin secretion/resistance and whether these indices can predict glucose intolerance (prediabetes and diabetes) at 1-year postpartum in women with GDM.

**Methods:**

We included 211 women with GDM and assessed 1,5-AG levels at 28–32 weeks of gestational age, at 6–8 weeks and at 1-year postpartum. In the context of changing body mass and insulin resistance, we also investigated 1,5-AG/BMI and 1,5-AG/HOMA-IR to adjust for prevailing BMI and HOMA-IR.

**Results:**

Circulating 1,5-AG levels and 1,5-AG/BMI were stable between pregnancy and 6–8 weeks postpartum but increased at 1-year postpartum (*p* < 0.001). 1,5-AG/HOMA-IR increased between pregnancy and the early postpartum and stabilized at 1-year postpartum (*p* < 0.001). In the perinatal period, 1,5-AG/HOMA-IR was related to a higher insulin-resistance adjusted insulin secretion index (ISSI-2), lower BMI and higher MATSUDA (all *p* ≤ 0.001). 1,5-AG/pre-pregnancy BMI during pregnancy and 1,5-AG/HOMA-IR at 6–8 weeks postpartum (both *p* ≤ 0.024) predicted a lower risk of glucose intolerance at 1-year postpartum, the latter remained significant after adjusting for HbA1c.

**Conclusions:**

These results suggest that 1,5-AG/HOMA-IR and 1,5-AG/pre-pregnancy BMI change in the perinatal period. They could provide useful information and may serve as markers of future glucose intolerance, and for early risk stratification in women after GDM.

**Supplementary Information:**

The online version contains supplementary material available at 10.1007/s40618-025-02768-1.

## Introduction

Gestational diabetes mellitus (GDM) is any degree of glucose intolerance with onset or first recognition during pregnancy that does not fulfill the criteria of overt diabetes [1]. GDM is characterized by increased insulin resistance and/or reduced secretion and is associated with an increased risk of future diabetes and cardiovascular risk [2]. Therefore, it is useful to identify markers that can characterize the pathophysiological features of GDM, predict future glucose intolerance, and provide early risk stratification.

The polyol, 1,5-anhydroglucitol (1,5-AG) is currently used in clinic as a non-traditional marker for assessing poor glucose control, reflecting hyperglycemic episodes above 10.0 mmol/L [3]. Because 1,5-AG is not metabolically active, its circulating levels are rather constant. The main route of 1,5-AG disposal is through urine excretion, with most of it being reabsorbed in the renal tubules [4, 5]. This reabsorption process is competitively inhibited by glucose when glycemia surpasses the threshold for glucosuria (> 10 mmol/L, not relevant for the present study), leading to 1,5-AG urinary excretion and a decrease in its circulating levels [6]. Reduced circulating 1,5-AG levels are indicative of previous (1-2-weeks) glucose concentrations above the renal threshold for glucosuria [7, 8].

Circulating 1,5-AG levels are reduced in patients with type-2 diabetes (T2D) [6, 9–11] and correlate with the functional ß-cell mass, with changes preceding diabetes onset and hyperglycemia. In the absence of such high glucose, 1,5-AG levels may reflect in vivo changes in the ß-cell mass per se [11]. First uncovered in mouse models [12], this association has been further corroborated in human cohorts including subjects with T2D or patients without diabetes but undergoing acute loss of ß-cells secondary to the resection of half of their pancreas [11]. Moreover, arginine-induced insulin secretion, as an in vivo estimate of the functional ß-cell mass [13], directly correlates with circulating levels of 1,5-AG [11]. As insulin secretion increases in response to increased insulin resistance, not necessarily associated with a higher functional ß-cell mass, it is necessary to investigate 1,5-AG levels related to BMI or prevailing insulin resistance to correctly estimate the functional ß-cell mass.

During pregnancy, 1,5-AG levels drop at 24–28 gestational age (GA) with a reduction of approximately 40% compared with non-pregnant women or pregnant women at ≤ 23 weeks gGA [14, 15]. At about one-month post-partum 1,5-AG seem to return to non-pregnant levels [16]. There are no data on the progression of this biomarker in the early to late postpartum. In addition, no study has investigated the associations between 1,5-AG levels and indices of insulin secretion and/or insulin resistance in pregnancy. Women with GDM have lower insulin secretion and/or resistance and their 1,5-AG levels are lower compared to those without GDM [16, 17]. In pregnant populations including women with GDM, 1,5-AG has been reported as a predictor of neonatal complications [18, 19]. Studies have demonstrated that lower 1,5-AG levels correlate with higher 1-hour post-OGTT glucose levels, suggesting that 1,5-AG may be a useful biomarker for identifying β-cell dysfunction in women with GDM [20, 21]. As the perinatal period is marked by pronounced changes in insulin resistance, the role of 1,5 AG adjusted for BMI or prevailing insulin resistance, i.e. 1,5-AG/BMI or 1,5-AG/HOMA-IR, respectively, could be relevant.

This study investigated the changes in 1,5-AG from the third trimester of pregnancy up to one year postpartum in women with GDM and correlated its serum levels to indices of insulin resistance and insulin secretion during the perinatal period. We also investigated 1,5-AG as a predictor of glucose intolerance at 1-year postpartum. To adjust for prevailing BMI or insulin resistance, we analyzed the role of 1,5-AG/BMI and 1,5-AG/HOMA-IR in predicting glucose intolerance.

## Materials and methods

The current study is a secondary analysis of the MySweetheart trial (NCT02872974). The detailed study protocol has been previously described [22]. The trial tested the effect of an interdisciplinary lifestyle and psychosocial intervention on improving metabolic and mental health outcomes in women with GDM during pregnancy up to 1-year postpartum between 2016 and 2021. The Human Research Ethics Committee of the Canton de Vaud (No. 2016 − 00745) approved the study protocol. Out of the 211 women included at baseline (28–32 weeks GA) (105 randomized to intervention and 106 to usual care), 39 women were excluded from this analysis (*n* = 16 at 6–8 weeks and *n* = 23 at the 1-year postpartum). Of those excluded, 82% (*n* = 32/39) were lost to follow-up, 12.8% (*n* = 5/39) had new pregnancies before the 1-year postpartum visit and 5.1% (*n* = 2/39) had their GDM diagnosis revised after group allocation. All included participants had 1,5-AG data at 6–8 weeks and 1-year postpartum. This consisted of 196 women at 6–8 weeks postpartum and 172 women at 1-year postpartum.

### GDM management and patient follow-up

We followed-up women in the usual care group according to the ADA and the Endocrine Society guidelines [1, 23]. Women were first seen at 28–32 weeks gestational age (GA) by a physician, or diabetes-specialist nurse who then followed them until delivery according to Swiss guidelines [24]. The detailed description of the intervention and usual-care groups of MySweetheart trial has been previously described [25, 26]. On top of the usual care, the intervention consisted of four clinical lifestyle sessions during pregnancy and four lifestyle and psychosocial visits in the postpartum, two peer support group workshop (one in pregnancy and one in the postpartum), and a bimonthly lifestyle coach support, mostly through telemedicine.

## Measures

### Metabolic health variables

Pre-pregnancy body mass was extracted from participants’ medical charts. We measured body mass and height at the first GDM visit and body mass at the end of pregnancy, at 6–8 weeks and 1-year postpartum using electronic scales (Seca^®^). Information on the need for glucose-lowering medical treatment (use of insulin and/or metformin) during pregnancy was extracted from maternal medical records. HbA1c was measured with a chemical photometric method (conjugation with boronate-Afinion^®^) at baseline and with a High-Performance Liquid Chromatography method (HPLC) in the postpartum [27]. At baseline we performed fasting measures of glucose and insulin before the initiation of any insulin treatment and at 6–8 weeks and 1-year postpartum, we performed a 75-g oral glucose tolerance test (oGTT). All women had their glucose-lowering medical treatment stopped at delivery. We defined glucose intolerance at 1-year postpartum (FPG ≥ 5.6 mmol/l or HbA1c ≥ 39 mmol/mol (≥ 5.7%) or 2-h glucose ≥ 7.8 mmol/l) according to the ADA criteria [1].

### Indices of insulin secretion or resistance

During the oGTT at 6–8 weeks postpartum, we measured glucose and insulin values at fasting and every 30 min over 2 h to calculate insulin secretion/sensitivity indices. The Homeostatic Model Assessment for Insulin Resistance (HOMA-IR) was used as a measure of insulin sensitivity [28]. Whole body insulin sensitivity was estimated with the MATSUDA index [29]. Absolute insulin secretion was estimated using Area under the Curve (AUC_ins/glu_) according to the trapezoidal rule [29]. To account for prevailing insulin resistance, the insulin resistance-adjusted insulin secretion was assessed using Insulin Secretion-Sensitivity Index-2 (ISSI-2), also known as disposition index [30]. At 28–32 weeks GA and at 6–8 weeks postpartum, we also calculated another index to characterise insulin secretion relative to the BMI, i.e. HOMA of ß-cell index (HOMA-B)/BMI.

### Measurement of 1,5-AG

We assessed circulating levels of 1,5-AG in samples obtained during the fasting state and stored at -80 °C. Measurements of 1,5-AG performed using a two-step enzymatic colorimetric assay (GlycoMark, NY) conducted on a FlexStation 3 Multimode microplate reader (Molecular Devices, San Jose). To mitigate potential interference from glucose, the samples were first treated with glucokinase to convert glucose into glucose-6-phosphate. The levels of 1,5-AG remain unaffected by factors such as meals or exercise, ensuring that variations in the sampling time of day do not impact this assessment [11, 31]. To characterize insulin secretion relative to the BMI and to the prevailing insulin resistance, we calculated two additional indices of 1,5-AG, i.e., 1,5-AG relative to BMI (1,5-AG/BMI) and 1,5-AG relative to HOMA-IR (1,5-AG/HOMA-IR).

### Socio-demographic and medical characteristics

Information on maternal socio-demographic characteristics including age, nationality/ethnic origin and educational level were collected during the first GDM visit. Data on medical characteristics including previous history of GDM, family history of diabetes, gravida, and parity were extracted from participants’ medical charts if available or were obtained from participants during the first GDM visit.

### Statistical analyses

All statistical analyses were performed with Stata/SE 15.1 (StataCorp LLC, TX, USA). Socio-demographic and medical characteristics were presented as either means (± standard deviation) or in frequency and percentages (%) where applicable (Table 1). Outcome and predictor variables including 1,5-AG, 1,5-AG/HOMA-IR, 1,5-AG/BMI, fasting glucose, 2 h glucose after oGTT, indices of insulin resistance (HOMA-IR, MATSUDA) and secretion (AUCins/glu), and relative insulin secretion (ISSI-2), HOMA-B/BMI) at at 28–32 weeks GA, 6–8 weeks postpartum were normally distributed.

**Table 1:** Baseline maternal socio-demographic and clinical characteristics of study participants

Variable	Mean ± SD
*N*	211
Age (year)	33.8 ± 4.4
GA at 28–32 weeks GA (weeks)	28.8 ± 2.4
BMI at 28–32 weeks GA (kg/m^2^)	29.6 ± 5.0
BMI at 28–32 weeks GA (kg/m^2^) (IQR)	29.1 (6.8)
GWG up to baseline visit (kg)	10.2 ± 5.8
HbA1c (%)	5.0 ± 0.33
Nationality/Ethnicity (n, %)	
Switzerland	62 (32.4)
Rest of Europe and North America	83 (43.4)
Asia and Oceania	23 (12.0)
Africa	14 (7.3)
Latin America	7 (3.7)
Others	2 (1.0)
Education level^a^ (n, %)	
Compulsory school incomplete^b^	2 (1.1)
Compulsory school achieved	23 (13.0)
High school	19 (10.7)
General and vocational education	42 (23.7)
University	91 (51.4)
Glucose-lowering treatment in pregnancy (n, %)	
Yes	90 (42.6)
No	121 (57.4)
Parity (n, %)	
0	120 (56.9)
1	57 (27.0)
2	18 (8.5)
≥3	16 (7.6)
Gravida (n, %)	
1	88 (41.7)
2	50 (23.7)
≥3	73 (34.6)
GDM in previous pregnancy^c^ (n, %)	
Yes	25 (11.8)
Family history of diabetes^d^ (n, %)	
Yes	136 (64.4)

GDM denotes gestational diabetes mellitus; SD denotes standard deviation; GA denotes gestational age; BMI denotes body mass index, GWG denotes gestational body mass gain; IQR denotes Interquartile range

All values are expressed as mean and standard deviations or n, %

^a^34 participants had missing data on education

^b^In Switzerland, compulsory schooling lasts eleven years

^c^Only for women who had at least one previous pregnancy

^d^Family history of diabetes consists of those with first degree (e.g., mother, father, brother, sister, daughter, son) and second degree (at least 25% of genetic link that included grandparents, grandchildren, nephews, niece, half-brother, and half-sister) relationship of the participant

We performed a repeated measure analysis with Bonferroni correction to determine the changes in 1,5-AG and indices insulin resistance and secretion at 28–32 weeks GA, 6–8 weeks, and 1-year postpartum in GDM women with normal glucose tolerance, i.e., those who did not develop glucose intolerance at 1-year postpartum. A linear regression was performed to determine the cross-sectional associations between 1,5-AG and 1,5-AG/HOMA-IR with body mass, BMI and indices insulin resistance and secretion during pregnancy, at 6–8 weeks postpartum and 1-year postpartum in all GDM participants.

We then investigated the predictors of glucose intolerance at 1-year postpartum using logistic regression analyses. Potential predictors tested during pregnancy were measures of glucose metabolism, indices of insulin secretion and resistance derived by the fasting blood: 1,5-AG, 1,5-AG/HOMA-IR and 1,5-AG/pre-pregnancy BMI, whereas those at 6–8 weeks postpartum were the same predictors such as in pregnancy, but at the 6–8 weeks postpartum time point and three additional measures derived from the oGTT: 2 h glucose value after oGTT, AUC_ins/glu_ and ISSI-2. In addition, analyses regarding measures of 1,5 AG and 1,5 AG/(pre-pregnancy) BMI or 1,5-AG/HOMA-IR were also adjusted for HbA1c. In all analyses, both predictors and outcomes were similar in the intervention and usual care groups and effect sizes of the relationship between 1,5-AG and all outcomes were similar if we did the analyses for the total sample or restricted them only to the control group. Therefore, we pooled participants in both groups to increase the sample size and adjusted for group allocation in all analyses. We also investigated the changes in 1,5-AG and indices of insulin resistance and secretion during pregnancy, at 6–8 weeks and 1-year postpartum in women with glucose intolerance, i.e., those 60 women who developed prediabetes/diabetes at 1-year postpartum using repeated measure analysis with Bonferroni correction (Supplementary Table 1). All statistical significances were two sided and accepted at *p* < 0.05.

## Results

We included 211 women with GDM at 28–32 weeks GA during pregnancy (baseline). Of these, 93% (196) and 82% (172) completed the 6–8 weeks and 1-year postpartum visits respectively. The mean pre-pregnancy body mass and BMI were 80.0 ± 14.71 kg and 25.91 ± 5.46 kg/m^2^ respectively (Table 1).

### Changes in 1,5-AG and indices of insulin resistance and secretion

Table 2 shows the changes in body mass, indices of insulin resistance and secretion, and 1,5-AG levels during pregnancy, 6–8 weeks, and 1-year postpartum in the 102 women with GDM who did not develop glucose intolerance at 1-year postpartum. Levels of 1,5-AG and 1,5-AG/BMI remained stable between 28-32 weeks GA and 6–8 weeks postpartum but increased at 1-year postpartum (overall *p* < 0.001), while 1,5-AG/HOMA-IR increased between pregnancy and 6–8 weeks postpartum, then stabilized (overall *p* < 0.001). HOMA-IR and HOMA-B/BMI decreased between pregnancy and 6–8 weeks postpartum along with expected insulin resistance, while HOMA-IR increased again at 1-year postpartum (overall p value for all *p* < 0.001). Post-delivery, ISSI-2 and MATSUDA decreased whereas AUC_ins/glu_ increased between 6 and 8 weeks and at 1-year postpartum (all *p* ≤ 0.009). When the analysis was restricted to the 60 participants who developed glucose intolerance at 1-year postpartum (Supplementary Table 1), we observed similar changes (overall *p* < 0.001).

**Table 2:** Changes in body mass, 1,5 AG and indices of insulin resistance and secretion in women with normal glucose tolerance at 1-year postpartum

Variable	(A) 28–32 weeks GA (*n* = 131) Mean ± SD	(B) At 6–8 weeks pp (*n* = 122) Mean ± SD	(C) At 1-yearpp (*n* = 102) Mean ± SD	Overall *P* value	*P* value B vs. A	*P* value C vs. A	*P* value B vs. C
1,5-AG (µg/ml)	10.89 ± 5.2	11.69 ± 5.3	16.21 ± 5.8	< 0.001	0.550	< 0.001	< 0.001
HOMA-IR	3.17 ± 1.69	1.74 ± 1.5	2.41 ± 1.4	< 0.001	< 0.001	0.002	0.007
1,5-AG/HOMA-IR	3.93 ± 2.78	10.02 ± 8.01	8.94 ± 5.99	< 0.001	< 0.001	< 0.001	0.607
1,5-AG/BMI	0.40 ± 0.20	0.45 ± 0.21	0.65 ± 0.26	< 0.001	0.172	< 0.001	< 0.001
HOMA-B/BMI	1.91 ± 0.81	1.07 ± 0.88	1.44 ± 0.71	< 0.001	< 0.001	< 0.001	0.004
ISSI-2		2.79 ± 1.02	2.42 ± 0.75	0.009			0.009
MATSUDA index		7.90 ± 3.9	5.76 ± 2.9	0.002			0.001
AUCins/glu		0.40 ± 0.1	0.50 ± 0.2	0.005			0.005
Body mass (kg)	74.15 ± 12.8	71.41 ± 13.6	68.83 ± 14.8	0.001	0.182	0.002	0.444
BMI (kg/m^2^)	27.39 ± 4.3	26.48 ± 4.6	25.54 ± 4.6	0.001	0.208	0.001	0.358

Data for the intervention and control group were pooled and adjusted for group allocation. BMI denotes body mass index; SD denotes standard deviation; pp denotes postpartum; 1,5-AG denotes 1,5-Anhydroglucitol

HOMA-IR denotes Homeostatic Model Assessment for Insulin Resistance; ISSI-2 denotes; Insulin Secretion-Sensitivity Index-2; AUC denotes Area under the Curve

Data is presented as mean ± standard deviation. P-values are derived from ANOVA with post-hoc Bonferroni correction

Sample sizes for body mass and BMI data are *n* = 2 (pregnancy) up to *n* = 7 (1-year pp) higher

### Associations between 1,5-AG during pregnancy and postpartum and body mass, BMI, and indices of insulin secretion/resistance

Table 3 shows the cross-sectional associations between 1,5-AG and 1,5-AG/HOMA-IR during pregnancy and postpartum with body mass, BMI and indices of insulin resistance and secretion in all participants. At 28–32-week GA, 1,5-AG was positively associated with body mass (*p* = 0.033), but not with other indices. At 1-year postpartum, 1,5-AG correlated positively with higher body mass and BMI and negatively with elevated fasting glucose and lower MATSUDA, as well as lower ISSI-2 (all *p* ≤ 0.034). During pregnancy, 1,5-AG/HOMA-IR was related to more favorable metabolic health outcomes such as lower body mass and fasting glucose (all *p* ≤ 0.001). At 6–8 weeks and 1-year postpartum it was further associated with increased MATSUDA and ISSI-2 and lower absolute AUC_ins/glu_ (all *p* ≤ 0.001).

**Table 3:** Cross-sectional associations between 1,5-AG during pregnancy and in the postpartum with body mass, BMI and markers of insulin resistance and secretion in all patients (*n* = 196)

Variable	β-coefficient	95% CI	*P* value
**1,5-AG at 28–32-week GA**			
Body mass (kg)	0.33	-0.05-0.71	0.089
Fasting glucose (mmol/L)	-0.06	-0.02-0.06	0.305
**1**,**5-AG/HOMA-IR at 28–32-week GA**			
Body mass (kg)	-1.55	-2.32, -0.87	< 0.001
Fasting glucose (mmol/L)	-0.06	-0.08, -0.04	< 0.001
**1**,**5-AG at 6–8 weeks pp**			
Body mass (kg)	0.37	0.03–0.74	0.033
BMI (kg/m^2^)	0.11	-0.001-0.23	0.053
Fasting glucose (mmol/L)	-0.01	-0.01-0.02	0.985
2 h glucose after OGTT (mmol/L)	-0.01	-0.04-0.03	0.954
MATSUDA index	-0.08	-0.18-0.01	0.105
ISSI-2	-0.04	-0.04-0.01	0.285
AUC_ins/glu_	0.05	-0.02-0.01	0.062
**1**,**5-AG/HOMA-IR at 6–8 weeks**			
Body mass (kg)	-0.65	-0.95, -0.35	< 0.001
BMI (kg/m^2^)	-0.24	-0.35, -0.14	< 0.001
Fasting glucose (mmol/L)	-0.04	-0.05, -0.02	< 0.001
2 h glucose after OGTT (mmol/L)	-0.03	-0.05-0.002	0.073
MATSUDA index	0.38	0.31–0.45	< 0.001
ISSI-2	0.06	0.04–0.09	< 0.001
AUC_ins/glu_	-0.01	-0.15, -0.006	< 0.001
**1**,**5-AG at 1-year pp**			
Body mass (kg)	0.57	0.19–0.95	0.003
BMI (kg/m^2^)	0.20	0.07–0.34	0.003
Fasting glucose (mmol/L)	0.017	0.001–0.03	0.032
2 h glucose after OGTT (mmol/L)	0.17	-0.04-0.05	0.938
MATSUDA index	-0.08	-0.17, -0.006	0.034
ISSI-2	-0.03	-0.05, -0.01	0.005
AUC_ins/glu_	0.02	-0.06-0.01	0.634
**1**,**5-AG/HOMA-IR at 1-year pp**			
Body mass (kg)	-1.36	-1.76, -0.96	< 0.001
BMI (kg/m^2^)	-0.52	-0.66, -0.37	< 0.001
Fasting glucose (mmol/L)	-0.03	-0.05, -0.02	< 0.001
2 h glucose after OGTT (mmol/L)	-0.08	-0.12, -0.03	0.001
MATSUDA index	0.35	0.28–0.41	< 0.001
ISSI-2	0.05	0.03–0.08	< 0.001
AUC_ins/glu_	-0.02	-0.03, -0.01	< 0.001

1,5AG denotes 1,5-anhydroglucitol; pp denotes postpartum; GA denotes gestational age; HOMA-IR denotes Homeostatic Model Assessment for Insulin Resistance; ISSI-2 denotes; Insulin Secretion-Sensitivity Index-2; AUC denotes Area under the Curve. Regression model is adjusted for group allocation

### Prediction of glucose intolerance at 1-year postpartum

Predictors in pregnancy and in the early postpartum for glucose intolerance at 1-year postpartum are shown in Table 4. Neither 1,5-AG levels during pregnancy and at 6–8 weeks postpartum nor 1,5-AG/BMI at 6–8 weeks postpartum did predict glucose intolerance at 1-year postpartum. However, 1,5-AG/pre-pregnancy BMI predicted lower odds of glucose intolerance at 1-year postpartum (*p* = 0.015). Similarly, 1,5-AG/HOMA-IR at 6–8 weeks postpartum predicted a lower risk of glucose intolerance at 1-year postpartum (*p* = 0.012), while its prediction during pregnancy was close to significance (*p* = 0.058). HOMA-B/pre-pregnancy BMI predicted an increased risk of glucose intolerance at 1-year postpartum (*p* = 0.024). When we adjusted for HbA1c, the results for 1,5-AG levels, 1,5-AG/HOMA-IR and HOMA-B/BMI remained significant.

**Table 4:** Prediction of glucose intolerance at 1-year postpartum by 1,5 AG and indices of insulin resistance and secretion (*n* = 172)

Variable	OR	95% CI	*P* value
**28–32 weeks GA**			
1,5-AG (µg/ml)	0.98	0.92–1.04	0.672
1,5-AG/HOMA-IR	0.87	0.76–1.02	0.058
1,5-AG/pre-pregnancy BMI	0.13	0.02–0.68	0.015
HOMA-B/BMI	1.61	1.06–2.45	0.024
**At 6–8 weeks pp**			
1,5-AG (µg/ml)	1.03	0.97–1.08	0.265
1,5-AG/HOMA-IR	0.92	0.86–0.98	0.012
1,5-AG/BMI	0.69	0.16-3.00	0.626
Fasting glucose (mmol/L)	2.16	1.30–3.59	0.003
2 h glucose (mmol/L)	1.49	1.15–1.93	0.002
HOMA-B/BMI	1.37	0.911–2.06	0.130
AUCins/glu	4.61	0.76–27.89	0.096
ISSI-2	0.39	0.23–0.66	< 0.001

1,5-AG denotes 1,5-anhydroglucitol; pp denotes postpartum; GA denotes gestational age; BMI denotes Body mass index

HOMA-IR denotes Homeostatic Model Assessment for Insulin Resistance; ISSI-2 denotes; Insulin Secretion-Sensitivity Index-2; AUC denotes Area under the Curve

Regression model is adjusted for group allocation

## Discussion

We investigated changes in circulating 1,5 AG, a potential marker of the functional ß-cell mass and insulin secretion capacity, at 28–32 weeks GA during pregnancy up to 1-year postpartum in women with normal glucose tolerance. Pregnancy was associated with rather low levels of 1,5-AG, while being restored post-delivery to the normal range between the 6–8 weeks and 1-year postpartum. The perinatal period is marked by pronounced changes in body mass and insulin resistance. 1,5-AG/HOMA-IR did not change in the postpartum, suggesting that the increase in the functional ß-cell mass (elevation of 1,5-AG) was primarily the consequence of increased insulin resistance (HOMA-IR) between the early and late postpartum. In the perinatal period, higher 1,5-AG/HOMA-IR was related to a healthier metabolic profile. 1,5-AG/pre-pregnancy BMI during pregnancy and 1,5-AG/HOMA-IR at 6–8 weeks postpartum predicted a lower risk of glucose intolerance at 1-year postpartum, in contrast to 1,5-AG. Thus, 1,5-AG/BMI pre-pregnancy index used during pregnancy does not need fasting blood draws and could be used as novel index for risk stratification in women with GDM. Although BMI and HOMA-IR provide practical and clinically relevant adjustments for 1,5-AG, pregnancy and postpartum involve additional metabolic adaptations. In our study, complementary OGTT-derived indices of insulin sensitivity and β-cell function supported the robustness of the 1,5-AG-based associations, highlighting their physiological significance.

Our findings are in line with previous studies that show decreased levels of 1,5-AG during pregnancy. Because 1,5-AG is a non-metabolized polyol with stable whole-body pool, this might just be contributed by a diluting effect secondary to changes in body fluid volumes that occur during this period [32]. In our cohort, 1,5-AG levels in the late postpartum were higher compared to those during pregnancy, consistent with prior studies in the perinatal period [16]. The reduced levels of 1,5-AG during pregnancy could also be explained by the increased glomerular filtration and changing urinary glucose excretion threshold [33]. When we adjusted for HOMA-IR, levels of 1,5-AG/HOMA-IR were lower in pregnancy and increased at 6–8 weeks postpartum but remained stable up to 1-year postpartum. In the postpartum period, higher 1,5-AG/HOMA-IR values were positively associated with MATSUDA and ISSI-2 and negatively associated with fasting and 2-h glucose levels, indicating more effective β-cell mass expansion with increased secretory adaptation relative to insulin resistance.

In the postpartum period, higher 1,5-AG levels correlated with higher body mass, BMI, fasting glucose as well as lower insulin sensitivity and insulin resistance-adjusted insulin secretion or disposition index. Overall, 1,5-AG reflected robust ß-cell state and insulin secretion, in response to increased insulin resistance. Moreover, 1,5-AG/HOMA-IR was related to a higher ISSI-2 in the postpartum, and thus could serve as a useful surrogate marker of adjusted insulin secretion. In addition, 1,5-AG/HOMA-IR was related to improved glucose control. This indicates that 1,5-AG/HOMA-IR could serve as a marker for assessing insulin secretion adjusted to insulin-resistance in these women.

In our cohort, 1,5-AG did not predict glucose intolerance at 1-year postpartum. However, both 1,5-AG/pre-pregnancy BMI during pregnancy and 1,5-AG/HOMA-IR at 6–8 weeks postpartum predicted a lower risk of future glucose intolerance. When we adjusted for HbA1c, the results regarding 1,5-AG/HOMA-IR remained significant. 1,5-AG levels are particularly high in obese subjects without diabetes who can cope with the increased insulin resistance and maintain glucose tolerance in line with a putative expansion of their ß-cell mass [11, 34]. By extension, the present study shows that women with GDM presenting lower insulin-resistance- or BMI-adjusted 1,5-AG levels are more likely to develop glucose intolerance at 1-year postpartum. Therefore, a simple assessment of circulating 1,5-AG/pre-pregnancy BMI could potentially be used in pregnancy as a marker of future glucose intolerance and or risk stratification. Compared to other conventional markers, 1,5-AG/BMI needs neither fasting measures nor ß-cell stimulus administration.

From a clinical perspective, our findings suggest that 1,5-AG–based indices may complement existing postpartum screening strategies. Because measurement of 1,5-AG is inexpensive, standardized, and does not require fasting, incorporating 1,5-AG or 1,5-AG/pre-pregnancy BMI during late pregnancy follow-up could help stratify women based on their risk of future glucose intolerance. For example, women with low 1,5-AG/HOMA-IR or 1,5-AG/pre-pregnancy BMI values, indicating a lower relative β-cell reserve, could be prioritized for early postpartum glucose testing or preventive lifestyle programs, while those with higher values may require less intensive monitoring. Such integration into clinical pathways would provide a non-invasive, scalable approach to risk stratification, pending confirmation in larger and more diverse cohorts.

The strengths of this study include its prospective design and longitudinal follow-up up to 1-year postpartum of a multi-ethnic cohort of women at high metabolic risk. The lack of a control group in the postpartum, i.e., women without GDM is a limitation. As the values of predictors and outcomes and their effect sizes were similar for both the intervention and the control group, we pooled participants and adjusted for group allocation in all analyses. We did not include HOMA-B/HOMA-IR in our analysis because its calculation as a measure of disposition index is controversial [35]. In our analyses predicting future glucose intolerance, we adjusted for HbA1c, but not fasting plasma glucose, as it is included in measures such as HOMA-IR. BMI and HOMA-IR were used to adjust 1,5-AG levels for adiposity and insulin resistance, but they do not fully capture the complex metabolic changes that occur during pregnancy and postpartum. However, our analyses also included OGTT-derived indices of insulin sensitivity and β-cell function, which help mitigate this limitation and strengthen the physiological interpretation of the 1,5-AG-based findings. Another limitation is the lack of concurrent measures of renal function, such as serum creatinine or estimated glomerular filtration rate. We did not assess renal function by measurements of creatinine clearance, which would have enabled a more accurate evaluation of renal contributions to circulating 1,5-AG. Given that gestational hyperfiltration and a lower renal glucose threshold may transiently reduce 1,5-AG levels, future studies should investigate measures of serum creatinine or estimated glomerular filtration rate to help clarify the contribution of renal function to 1,5-AG dynamics during the perinatal period. Although dietary intake and lactation-related metabolic changes could influence 1,5-AG concentrations, these were not assessed in the present analysis and are beyond the scope of this study. As this study is focused on 1,5-AG and its derived indices, other glycemic or metabolic biomarkers were not evaluated. While 1,5-AG provides unique information about short-term glycemic fluctuations and functional β-cell mass compensation, as observed in obese healthy subjects [11] it does not capture the full spectrum of metabolic alterations that contribute to postpartum glucose intolerance. Future studies incorporating multi-marker panels could enhance predictive accuracy and clinical applicability. While our findings provide insight into the short-term (1-year) trajectory of 1,5-AG and its relationship with postpartum glucose intolerance, longer follow-up is needed to determine whether these associations persist and predict later development of type 2 diabetes or cardiometabolic disease. Prospective studies extending several years postpartum are needed to establish the clinical utility of 1,5-AG–based indices for long-term metabolic risk stratification in women with prior GDM.

## Conclusions

In this cohort of women with GDM, levels of 1,5-AG increased between the early and late postpartum and were higher compared to pregnancy. However, 1,5-AG/HOMA-IR did not change in the postpartum. This suggests that the known increase in insulin resistance between the early and late postpartum might have led to a compensatory increase in the functional ß-cell mass. 1,5-AG/HOMA-IR was lower in pregnancy compared to the postpartum, which is probably due to higher body fluid volumes during gestation with dilution of the overall 1,5-AG pool, along with the potential changes in increased renal clearance. During the perinatal period, 1,5-AG/HOMA-IR was related to established indices of insulin-resistance-adjusted insulin secretion such as ISSI-2. This study also shows that women with GDM with lower BMI-adjusted 1,5-AG levels are more likely to develop glucose intolerance at 1-year postpartum. Therefore, a simple non-fasting assessment of circulating 1,5-AG/pre-pregnancy BMI could potentially be used in pregnancy as a reliable marker of future glucose intolerance and or risk stratification.

## Electronic Supplementary Material

Below is the link to the electronic supplementary material.

Supplementary Material 1

## Data Availability

The datasets generated and/or analyzed during the current study are not publicly available as they are clinical data but are available from the corresponding author on reasonable request.
