# Supplementary material for: Circulating 1,5-anhydroglucitol levels combined with pre-pregnancy BMI is a simple predictive marker of future glucose intolerance in women with gestational diabetes
Source: J Endocrinol Invest. 2025 Dec 12;49(4):969–78. doi: 10.1007/s40618-025-02768-1 (PMC13053329; doi:10.1007/s40618-025-02768-1)
Supplement: Supplementary file 1 — Supplementary Material 1 [file 40618_2025_2768_MOESM1_ESM.docx]

**Supplementary Table 1:** Changes in body mass, 1,5-AG and indices of insulin resistance and secretion in women with glucose intolerance at 1-year pp (n=60)

| Variable | **(A)**  **28-32 weeks GA**  **(n=60)** | **(B)**  **At 6-8 weeks pp**  **(n=57)** | **(C)**  **At 1-year**  **pp**  **(n=58)** | Overall  P value | P value B vs A | P value C vs A | P value B vs C |
| --- | --- | --- | --- | --- | --- | --- | --- |
|  | **Mean±SD** | **Mean±SD** | **Mean±SD** |  |  |  |  |
| 1,5-AG (μg/ml) | 11.03±5.6 | 12.69±7.5 | 18.06±6.4 | <0.001 | 0.121 | <0.001 | <0.001 |
| HOMA-IR | 4.42±2.4 | 2.69±2.1 | 4.73±2.8 | <0.001 | <0.001 | 0.560 | <0.001 |
| 1,5-AG/HOMA-IR | 3.05±2.9 | 7.25±6.2 | 5.41±4.0 | <0.001 | <0.001 | 0.012 | 0.074 |
| 1,5-AG/BMI | 0.39±0.2 | 0.43±0.2 | 0.62±0.2 | <0.001 | 0.587 | <0.001 | <0.001 |
| HOMA-B/BMI | 2.18±0.9 | 1.25±0.9 | 1.86±1.0 | <0.001 | <0.001 | 0.188 | 0.001 |
| ISSI-2 |  | 2.06±0.5 | 1.41±0.5 | <0.001 |  |  | <0.001 |
| MATSUDA |  | 5.63±2.9 | 2.66±1.3 | <0.001 |  |  | <0.001 |
| AUCins/glu |  | 0.45±0.2 | 0.64±0.3 | 0.003 |  |  | 0.004 |
| Body mass (kg) | 76.24±14.6 | 79.65±15.6 | 79.76±17.0 | 0.097 | 0.227 | 0.312 | 0.865 |
| BMI (kg/m^2^) | 28.16±5.2 | 29.54±5.5 | 29.49±6.0 | 0.058 | 0.131 | 0.252 | 0.564 |

Data for the intervention and control group were pooled and adjusted for group allocation; BMI denotes body mass index; SD denotes standard deviation; pp denotes postpartum; 1,5-AG denotes 1,5-Anhydroglucitol; AH index denotes 1,5‑Anhydroglucitol × glycated hemoglobin A1c/100.

HOMA-IR denotes Homeostatic Model Assessment for Insulin Resistance; ISSI-2 denotes; Insulin Secretion-Sensitivity Index-2; AUC denotes Area under the Curve

Data is presented as mean±standard deviation. P-values are derived from ANOVA with post-hoc Bonferroni correction.
